# ChromTag: an interactive R-shiny platform for analysis and visualization of CUT&Tag and ChIP-seq peak profiling data

**DOI:** 10.3389/fbinf.2026.1805772

**Published:** 2026-06-25

**Authors:** Siwen Xu, Qingyan Zou, Rui Shi, Jieru Huang, Zixiao Lu, Jin Yang

**Affiliations:** 1 School of Medical Information and Engineering, Guangdong Pharmaceutical University, Guangzhou, China; 2 Guangdong Province Precise Medicine Big Data of Traditional Chinese Medicine Engineering Technology Research Center, Guangdong Pharmaceutical University, Guangzhou, China; 3 School of Pharmacy, Guangdong Pharmaceutical University, Guangzhou, China; 4 School of Medical Information Engineering, Guangzhou University of Chinese Medicine, Guangzhou, China

**Keywords:** chromatin profiling, Cut&Tag, epigenomic analysis, gene regulation, R shiny web application, visualization platform

## Abstract

Chromatin profiling technologies such as CUT&Tag and ChIP-seq have greatly advanced epigenomic research by enabling genome-wide mapping of histone modifications and transcription factor binding, providing key insights into gene regulation, cellular differentiation, and disease mechanisms. However, interpreting these datasets remains difficult for many researchers due to the technical expertise required for data processing and analysis. To address this limitation, we developed ChromTag, an interactive web-based application built with R Shiny for the comprehensive exploration and visualization of CUT&Tag and other epigenomic profiling datasets. Using a modular workflow, ChromTag performs differential peak detection, assigns peaks to nearby genes based on user-defined genomic windows, and supports ORA-based GO and KEGG enrichment analysis and preranked GSEA using gene-level summaries derived from peak annotation. The platform separates upregulated and downregulated regions to distinguish activated from repressed regulatory pathways and incorporates motif enrichment analysis to highlight transcription factors that may cooperate with chromatin modifications to influence gene expression. ChromTag currently supports human, mouse, and *Drosophila* datasets, and provides extensive visualization options such as volcano plots, PCA, heatmaps, and genomic peak profiles. By bridging preprocessed peak count matrices with interactive visualization and exploratory functional interpretation, ChromTag provides a practical and accessible downstream analysis solution for chromatin profiling data.

## 1 Introduction

Chromatin modifications, including histone modifications and DNA methylation, are fundamental mechanisms that regulate gene expression and coordinate diverse biological processes such as development, differentiation, and disease progression (Policarpi et al., 2024). Deciphering these regulatory layers has become essential for understanding how genetic information is dynamically interpreted within specific cellular contexts.

To investigate chromatin-associated regulation, Chromatin Immunoprecipitation Sequencing (ChIP-seq) and Cleavage Under Targets and Tagmentation (CUT&Tag) have been established as two major experimental approaches in epigenomics (Abbasova et al., 2025). ChIP-seq maps genome-wide protein–DNA interactions by enriching DNA fragments bound to histone marks or transcription factors, followed by high-throughput sequencing (Park, 2009). While it has significantly advanced chromatin biology, the method requires large input material, involves complex workflows, and often suffers from high background noise, limiting its applicability to scarce samples.

CUT&Tag provides an efficient alternative that integrates target recognition and sequencing adapter insertion into a single reaction through a transposase–antibody fusion system (Kaya-Okur et al., 2019). This approach minimizes sample requirements, reduces experimental steps, and enhances the signal-to-noise ratio, thereby enabling high-resolution profiling of histone modifications and transcription factor binding from small cell populations. The continuous accumulation of ChIP-seq and CUT&Tag data has greatly expanded our ability to map chromatin landscapes across different tissues, cell types, and pathological states (Hsieh et al., 2022). These datasets have revealed numerous context-specific regulatory elements, enhancer–promoter interactions, and chromatin state transitions, forming a key resource for biomarker discovery and disease mechanism studies (Kundaje et al., 2015).

Despite these advances, the analysis of user-generated data remains a major bottleneck. Most available computational tools require considerable programming expertise and fragmented workflows that hinder reproducibility. Command-line tools such as MACS2 and HOMER are widely used for peak calling and motif analysis (Feng et al., 2012; McLeay and Bailey, 2010), but they provide limited support for downstream interpretation. Meanwhile, public repositories such as ENCODE, ChIP-Atlas, and the Cistrome Data Browser offer curated datasets and basic visualization (de Souza, 2012; Zou et al., 2024; Zheng et al., 2019).

To facilitate downstream interpretation of preprocessed chromatin profiling data, we developed ChromTag, an R Shiny–based web platform for interactive exploration and visualization of CUT&Tag and ChIP-seq peak count matrices. ChromTag is designed for experimental researchers and early-stage bioinformatics users who have access to preprocessed peak count matrices, a common scenario in collaborative and pipeline-based epigenomic workflows. ChromTag integrates differential peak detection, gene annotation, and enrichment analyses within a modular and user-friendly framework. The platform allows users to identify condition-specific chromatin changes, explore their biological significance, and visualize the results using volcano plots, principal component analysis, and heatmaps. Furthermore, a built-in motif enrichment module facilitates the identification of transcription factors potentially cooperating with histone modifications. All results can be exported in standard formats (PDF, PNG, JPEG, CSV), enabling seamless data interpretation and sharing. ChromTag thus provides a practical and accessible solution for downstream epigenomic analysis, bridging preprocessed peak count matrices and exploratory functional interpretation.

## 2 Implementation

### 2.1 Home module

The Home Module serves as the entry point of ChromTag, providing a clear and organized interface for initiating the analysis workflow. It consists of three primary components: a brief platform introduction, a data upload section, and a real-time data preview panel.

To help users explore the platform’s functionality, ChromTag provides preloaded example datasets consisting of H3K27ac profiles derived from human A549 cell lines treated with dexamethasone for 0 h and 4 h. These datasets are available from the ENCODE database (ENCSR783SNV and ENCSR543ZVZ) and GEO database (GSE91337 and GSE91282) (a. ENCODE Project Consortium, 2012). The raw FASTQ files of these datasets were previously processed using standard bioinformatics workflows, including read alignment, peak calling, and construction of a joint peak set across all samples, to generate count matrices compatible with ChromTag. Specifically, read counts were summarized over merged peak regions, producing a comma-separated values (.csv) file in which the first three columns specify genomic coordinates (chromosome, start, and end) and the remaining columns contain sample-specific read counts. These preprocessed count tables, which jointly define peaks across all samples, serve as the accepted input format for ChromTag analysis modules.

Users may also upload their own preprocessed datasets following the same standardized format. Each uploaded. csv file must contain genomic coordinates in the first three columns and corresponding read count values in subsequent columns. To ensure reliable statistical inference, a minimum of three biological replicates per group is recommended. Analyses based on fewer replicates may result in reduced statistical power and unstable variance estimation, and should therefore be interpreted with caution.

**FIGURE 1:**
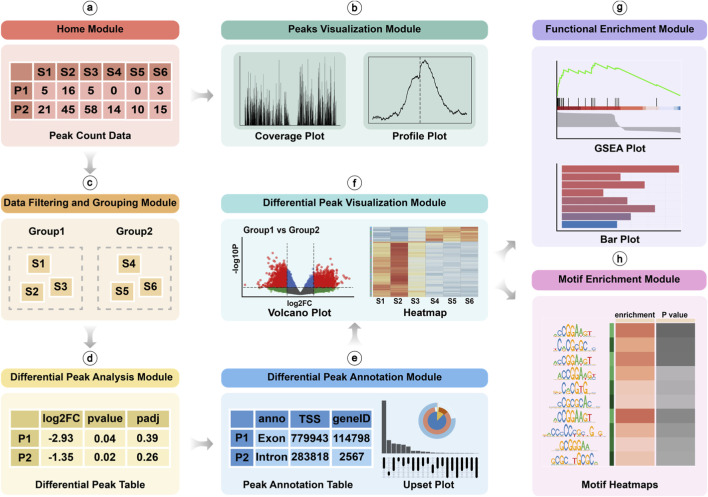
Overview of ChromTag functionality and analytical workflow. **(a)** Home module for data import and initialization. **(b)** Peaks Visualization module showing chromosome coverage and TSS profiles. **(c)** Data Filtering and Grouping module for preprocessing and sample organization. **(d)** Differential Peak Analysis module for identifying significant chromatin changes. **(e)** Differential Peak Annotation module linking peaks to nearby genes. **(f)** Differential Results Visualization module displaying volcano plots, MA plots, PCA, and heatmaps. **(g)** Functional Enrichment module for GO/KEGG ORA and preranked GSEA. **(h)** Motif Enrichment module identifying enriched transcription factor motifs.

After uploading, users can preview their dataset in an interactive table to confirm correct formatting and data integrity before proceeding to downstream analysis (Figure 1a).

### 2.2 Peaks Visualization module

The Peaks Visualization Module provides interactive tools for exploring the genome-wide distribution of identified peaks (Figure 1b). Two primary visualization types are available: the Chromosome Coverage Plot and the TSS Profile Plot. The Chromosome Coverage Plot displays peak density across chromosomes, whereas the TSS Profile Plot focuses on signal enrichment surrounding transcription start sites (TSSs). Both visualizations are implemented using the ChIPseeker package, a well-established toolkit for chromatin binding site analysis (Yu et al., 2015).

**FIGURE 2:**
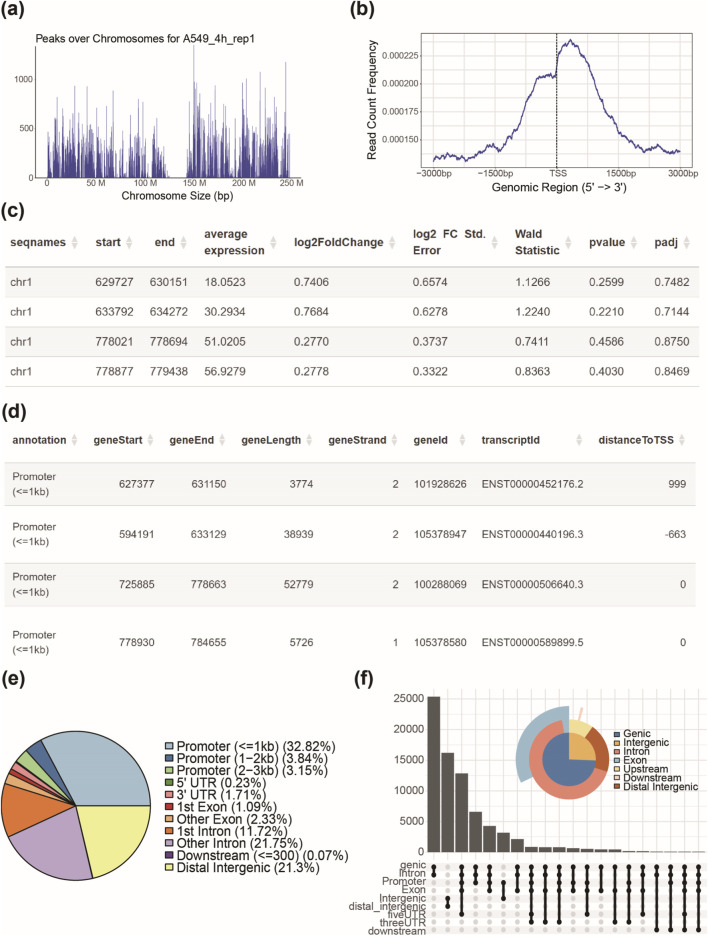
Visualization and downstream analysis of chromatin peaks using ChromTag. **(a)** Chromosome coverage plots showing the genomic distribution of peaks. **(b)** Profile plots illustrating read density around transcription start sites (TSS ±3 kb). **(c)** Differential peak analysis table. **(d)** Gene annotation table, showing annotations for peaks in (c). **(e)** Pie plot showing the distribution of annotated genomic features. **(f)** UpSet plot illustrating the intersections of annotated peak categories.

Before generating plots, users may assign custom colors to individual samples in the Color Assignment Panel. Once applied, these colors remain consistent across both visualization modes, improving readability and aiding comparative interpretation. Visualization of genome-wide peak distribution helps users identify chromosomes or regions with elevated or reduced peak density (Figure 2a), which may correspond to active regulatory features such as promoters or enhancers (Zhu et al., 2021). The TSS Profile Plot further provides a focused view of read intensity around gene promoters, allowing users to adjust the upstream and downstream window size (default ±3000 bp) based on experimental requirements (Figure 2b).

### 2.3 Data filtering and grouping module

The Data Filtering and Grouping Module performs essential preprocessing steps to ensure data quality and optimize downstream analysis. The filtering function removes genomic peaks with low read counts based on a user-defined threshold applied uniformly across all samples, independent of group assignments, retaining only those with sufficient sequencing coverage. This step effectively reduces data size and computational load, accelerating subsequent analyses while retaining peaks with sufficient read coverage for downstream analysis.

Users can interactively preview the dataset before and after filtering, allowing adjustment of the count threshold based on data-driven considerations during exploratory analysis. Once defined, filtering thresholds should be applied consistently and should not be modified *post hoc* to achieve desired outcomes.

Following filtering, the grouping function enables users to assign samples into defined experimental groups for comparative analysis (Figure 1c). Group names and sample memberships can be specified through an interactive interface, ensuring accurate organization of samples for differential peak detection. The system provides immediate visual feedback on the defined groups, and users can easily modify or reset group assignments as needed. This flexibility facilitates efficient data management and prepares the dataset for subsequent statistical testing and visualization steps.

### 2.4 Differential peak analysis module

The Differential Peak Analysis Module is a key analytical component of ChromTag, designed to identify statistically significant differences in peak intensities between two experimental groups (Figure 1d). It employs the DESeq2 framework for differential testing, where count data are modeled using a negative binomial distribution (Love et al., 2014). Normalization is handled internally by DESeq2 through the estimation of size factors to account for differences in sequencing depth and library composition across samples. It is important to note that normalization is a critical step in differential analysis, and improper normalization can lead to misleading results (Cabello-Solorzano et al., 2023; Coussement et al., 2024). We recommend users carefully consider the normalization process, especially when working with small datasets or samples with low sequencing depth, as this can significantly affect result interpretation.

For statistical inference, differential testing is performed at the peak level using the negative binomial model. To control for multiple testing, ChromTag supports both the Benjamini–Hochberg (BH) method, which controls the false discovery rate, and the *Holm* method, which controls the family-wise error rate through a stepwise adjustment (Ge et al., 2007; Thissen et al., 2002). The resulting adjusted p-values provide the primary statistical evidence for identifying differential peaks.

The module provides a set of user-configurable parameters through interactive panels, allowing users to explore results under predefined parameter settings. Users can specify the *Direction of Change*, selecting whether to focus on peaks with increased or decreased signal. Prior to DESeq2 modeling, ChromTag uses the filtered peak count matrix generated by the user-defined filtering step described above. This pre-analysis filtering is applied uniformly across all samples and is independent of group assignments and differential testing statistics. Its primary purpose is to remove peaks with insufficient read support, thereby reducing computational burden and facilitating downstream visualization and interpretation.

DESeq2 independent filtering is conceptually distinct from this user-defined pre-analysis filtering step. In ChromTag, DESeq2 independent filtering is provided as an advanced user-selectable option but is disabled by default. When explicitly enabled, DESeq2 performs independent filtering during result extraction using the mean normalized count of each peak prior to adjusted p-value calculation. This procedure identifies low-information peaks with very low mean signals that are unlikely to achieve statistical significance but may increase the multiple-testing burden. DESeq2 evaluates a range of mean-signal thresholds and automatically selects the threshold that maximizes, or nearly maximizes, the number of peaks with significant adjusted p-values.

Importantly, when DESeq2 independent filtering is enabled, peaks below the selected independent-filtering threshold are not removed from the final results table. Instead, their adjusted p-values may be reported as NA because they are excluded from the multiple-testing adjustment procedure. When the BH method is selected, the target significance level corresponds to the FDR cutoff; when the Holm method is selected, it corresponds to the significance cutoff for Holm-adjusted p-values. This parameter is used solely to optimize the optional independent filtering procedure, whereas final differential peaks are subsequently determined according to the user-specified adjusted p-value and log_2_ fold-change cutoffs. Consequently, when independent filtering is not applied, all peaks retained after the initial user-defined count filtering participate in the multiple-testing adjustment.

It is essential that all parameter settings, including significance thresholds, be determined based on data-driven insights during exploratory analysis. Arbitrary threshold selection to achieve statistical significance should be avoided, as it may introduce bias (Correia et al., 2024).

For two-group comparisons, the module performs direct differential testing. The output includes key statistical metrics such as base mean, adjusted p-value, log_2_ fold change, and normalized peak counts (Figure 2c). To avoid potential bias introduced by *post hoc* selection, no additional filtering is applied after differential testing (Ebrahimpoor and Goeman, 2021). Instead, results are presented based on statistically defined criteria, and users are encouraged to interpret differential peaks using adjusted p-values and effect sizes in a data-driven manner (Kroc and Olvera Astivia, 2022).

### 2.5 Differential peak annotation module

The Differential Peak Annotation Module annotates statistically significant differential peaks identified in the previous module and summarizes the results in both tabular and graphical formats (Figure 1e). This module leverages the ChIPseeker framework to assign each differential peak to annotated genomic features and to report the nearest annotated gene (Yu et al., 2015). The implementation and interactive visualization strategy of this module were adapted and extended from our previously published Linkage application, which also employed ChIPseeker-based genomic annotation for the interpretation of regulatory regions (Xu et al., 2025). Users can flexibly define upstream and downstream distance thresholds, enabling annotation of peaks located near promoters as well as peaks in distal genomic regions. However, it is important to note that ChIPseeker relies on predefined genomic annotations, which may not always capture the full complexity of chromatin regulation, especially in regions with sparse annotations or novel regulatory elements. Therefore, while ChIPseeker provides valuable insights for peak annotation, its reliance on available annotations may limit its ability to identify regulatory features in poorly annotated or unannotated regions.

Annotation results include promoter-TSS, exons, UTRs, introns, and intergenic regions. For each annotated peak, ChromTag retains the peak genomic coordinates and additionally reports nearest-gene annotation information, including the nearest annotated gene, gene coordinates, gene length, gene strand orientation, and the distance from the peak to the TSS. All annotations are presented in an interactive table that supports browsing and data export (Figure 2d).

To assist in biological interpretation, the module offers several visualization options. *Pie plots* and *bar plots* depict the proportion of peaks across genomic feature categories, while distance-to-TSS and *UpSet plots* illustrate spatial patterns and feature overlaps among annotated peaks (Figures 2e-f). These analyses provide biological context for the differential peaks identified earlier, bridging statistical findings with functional interpretation.

### 2.6 Differential peak visualization module

The Differential Peak Visualization Module enables intuitive exploration and interpretation of the results from differential peak analysis (Figure 1f). It provides a series of complementary visualizations, including volcano plots, MA plots, PCA plots, and heatmaps, which collectively reveal the overall landscape of differential chromatin signals and sample relationships.

**FIGURE 3:**
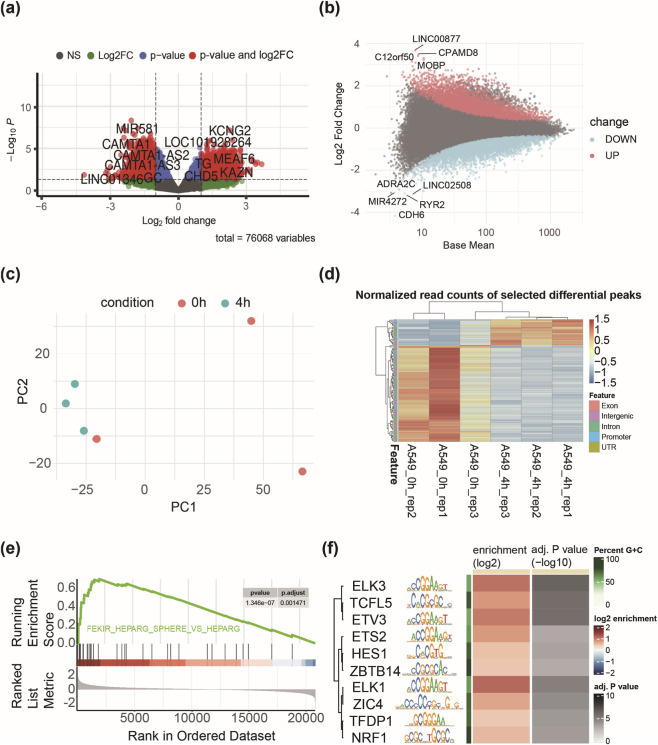
Examples of differential results visualization, functional enrichment analysis, and motif enrichment analysis generated by ChromTag. **(a)** Volcano plot of differential peaks. **(b)** MA plot of differential peaks showing the relationship between log_2_ fold change and mean normalized signal intensity. Selected peaks in (a) and (b) are labeled with their nearest annotated gene names to provide genomic context. **(c)** PCA plot showing sample clustering and replicate-level variability based on normalized peak signals. **(d)** Heatmap of normalized read counts for selected differential peaks. **(e)** GSEA enrichment plot based on a peak-to-gene summarized ranked gene list. **(f)** Motif enrichment heatmap for selected differential peak regions.

Among these, the volcano plot serves as the primary interactive visualization (Figure 3a). Users can define log_2_ fold-change and adjusted p-value thresholds to highlight differential peaks with significantly increased or decreased chromatin signals. In the volcano plot, selected peaks are labeled with their nearest annotated gene names to provide genomic context for peak-level differential signals. Gene labels are used solely as annotations of the corresponding peaks and do not represent gene-level differential testing results. The corresponding peak genomic coordinates and nearest-gene annotations are simultaneously displayed in an accompanying results table for downstream selection and export. The MA plot illustrates the relationship between log_2_ fold change and mean signal intensity (Figure 3b), providing an overview of both the magnitude and consistency of differential signals across the genome. Importantly, in addition to serving as a visualization tool, the MA plot can also be used as a diagnostic for assessing the effectiveness of normalization and overall data quality. Deviations from expected patterns may indicate potential violations of underlying assumptions, such as differences in signal distributions between conditions. Therefore, interpretation of MA plots should be performed with consideration of both the biological context and the characteristics of the input data.

The PCA plot allows users to evaluate global clustering patterns and sample variability (Figure 3c). By projecting samples into a low-dimensional space, it helps identify group separation and potential batch effects, thereby validating experimental consistency. Finally, the heatmap visualizes normalized signal patterns for selected differential peaks across samples (Figure 3d). Peaks passing the user-specified adjusted p-value and log_2_ fold-change thresholds are ranked by adjusted p-value, and up to the top 100 most statistically significant peaks are displayed in the heatmap. Rows represent peak regions defined by genomic coordinates, and row-scaled normalized read counts are used to highlight relative signal patterns across samples. Hierarchical clustering is applied using Euclidean distance and complete-linkage clustering to group peaks and samples with similar signal patterns. Genomic feature annotations are shown as row annotations to provide peak-level genomic context.

Together, these visualizations form a cohesive analytical interface that bridges statistical outputs and biological interpretation, allowing users to capture differential chromatin landscapes at both the global and feature-specific levels.

### 2.7 Functional enrichment module

The Functional Enrichment Module facilitates biological interpretation of differential peaks by identifying functional pathways and gene categories associated with genes assigned to peaks through genomic annotation (Figure 1g). It supports gene set analysis (GSA) through two complementary approaches: overrepresentation analysis (ORA) based on discrete peak-associated gene lists and gene set enrichment analysis (GSEA) based on a ranked gene-level list. ORA is performed using curated gene set resources such as Gene Ontology (GO) and Kyoto Encyclopedia of Genes and Genomes (KEGG), whereas GSEA is applied to a ranked gene-level list derived from peak-level log_2_ fold-change values after peak-to-gene summarization (Consortium, 2019; Kanehisa et al., 2017; Subramanian et al., 2005).

For GO and KEGG ORA, ChromTag performs enrichment analysis separately for genes associated with upregulated and downregulated differential peaks. Differential peaks are first assigned to their nearest annotated genes. Because multiple peaks may be assigned to the same gene, duplicate gene identifiers are collapsed so that each gene is counted only once in the ORA input. Genes associated with both increased-signal and decreased-signal differential peaks are classified as mixed-direction genes and are excluded from the direction-specific ORA gene lists by default to avoid ambiguous directional interpretation. These mixed-direction genes are displayed in a separate list in the Functional Enrichment Module for user inspection and transparency. Users may alternatively provide predefined gene lists for enrichment analysis. GO and KEGG ORA are implemented using clusterProfiler, and user-defined p-value and q-value cutoffs are used to filter the reported enriched terms or pathways. For peak-derived ORA, the background is defined as the set of unique genes associated with all annotated peaks retained after the initial read-count filtering step, because ORA results depend strongly on the choice of background gene set.

In contrast, GSEA is performed on a unified ranked gene-level list after excluding mixed-direction genes from the default input to avoid assigning an arbitrary direction to genes with conflicting peak-level signals. When multiple annotated peaks with the same direction of change are assigned to the same gene, duplicate gene identifiers are collapsed by selecting the peak with the smallest adjusted p-value as the representative peak. If multiple peaks share the same adjusted p-value, the peak with the largest absolute log_2_ fold change is selected. GSEA then evaluates enrichment across the entire ranked list, reducing reliance on predefined significance thresholds or gene subsets.

ChromTag integrates well-established gene set libraries, including Molecular Signatures Database (MSigDB) collections, and provides visualization of enrichment curves and leading-edge subsets based on the derived gene-level ranked list (Figure 3e) (Liberzon et al., 2011). Together, these complementary approaches enable users to explore functional patterns from both discrete and continuous perspectives.

### 2.8 Motif enrichment module

The Motif Enrichment Module investigates transcription factor (TF) binding motifs enriched within regions corresponding to upregulated and downregulated differential peaks, providing exploratory insights into sequence features associated with chromatin state changes (Figure 1h). By analyzing the sequence composition of selected differential peaks, the module identifies overrepresented motifs corresponding to candidate TFs that may be associated with the observed chromatin changes. Because motif occurrence alone does not establish functional TF binding or causal regulation, motif enrichment results should be interpreted in the context of the input data type. For TF ChIP-seq or CUT&Tag data, motif enrichment may support assessment of target motif recovery and suggest potential co-factor motifs, consistent with the concept that multiple TFs may co-occupy regulatory regions and act cooperatively *in vivo* (Sönmezer et al., 2021). For histone-mark data, motif enrichment may suggest TF families associated with chromatin state changes. Additional orthogonal evidence may be required for functional validation.

Motif analysis is performed on selected differential peak regions. By default, increased-signal and decreased-signal peaks passing the user-defined thresholds are ranked within each direction by adjusted p-value. If multiple peaks share the same adjusted p-value, they are further ranked by absolute log_2_ fold change. The top 200 peaks from each group are used for motif enrichment analysis. Users can adjust this number, refine the set of analyzed peaks, and select whether to include GC-content normalization or transcription factor clustering during visualization. Results are displayed as both a table and a heatmap, summarizing enrichment ratios, *p*-values, *q*-values, and motif-specific statistics (Figure 3f).

By comparing motif enrichment between upregulated and downregulated differential peaks, this module highlights candidate TF motifs associated with condition-dependent chromatin changes. Together with peak annotation and functional enrichment results, motif enrichment provides an exploratory view of potential regulatory factors while emphasizing that motif occurrence alone does not prove functional TF binding, target-gene regulation, or causal transcriptional control.

## 3 Results

### 3.1 Case study

To demonstrate the functionality and appropriate usage of ChromTag in a standard differential analysis setting, we conducted a case study using publicly available human ChIP-seq datasets for H3K27ac histone modification. The datasets were obtained from the ENCODE project, including Experiment ENCSR783SNV (GEO: GSE91337) representing A549 cells treated with 100 nM dexamethasone for 0 h, and Experiment ENCSR543ZVZ (GEO: GSE91282) representing treatment for 4 h (a. ENCODE Project Consortium, 2012). Each condition includes three biological replicates, forming a more appropriate experimental design for differential chromatin analysis.

This study design, based on the same histone modification under two conditions, allows for a more appropriate application of normalization-based differential analysis compared to comparisons across distinct chromatin marks. As such, it better satisfies the assumptions underlying methods such as DESeq2 and provides a more reliable framework for interpretation.

After preprocessing, peaks were jointly defined across all samples and used to construct a unified peak count matrix. Differential peak analysis was then performed using the DESeq2 framework (Love et al., 2014). Results were evaluated by jointly considering adjusted p-values and log_2_ fold changes, rather than relying on arbitrary thresholds alone.

Visualization results further support the appropriateness of the analysis. In particular, the MA plot shows a generally balanced distribution of signals across conditions, suggesting that normalization assumptions are reasonably satisfied and that the data are suitable for differential analysis. Downstream annotation and functional enrichment analyses enabled biological interpretation of the identified differential peaks. Enriched pathways, such as calcium signaling and axon guidance, suggest potential regulatory roles of H3K27ac in response to dexamethasone treatment (Figure 3).

Overall, this case study demonstrates that ChromTag can be applied in a statistically appropriate and biologically interpretable manner when underlying assumptions are reasonably satisfied, while also highlighting the importance of careful experimental design and result interpretation in differential chromatin analysis.

### 3.2 Comparison with other tools

To evaluate the performance and usability of ChromTag, we compared it with two established tools for epigenomic data analysis, MicroScope and ChIPdig (Khomtchouk et al., 2016; Esse, 2019).

MicroScope supports the input of raw count matrices but does not retain genomic coordinate information for peaks, which limits its ability to perform peak annotation or to associate peaks with functional genomic regions. ChIPdig, in contrast, focuses primarily on data reformatting and basic visualization. Its workflow requires multiple manual upload steps and lacks automatic integration between modules, which can extend the overall runtime to several hours. Additionally, it does not support downstream analyses such as functional enrichment or motif discovery.

**FIGURE 4:**
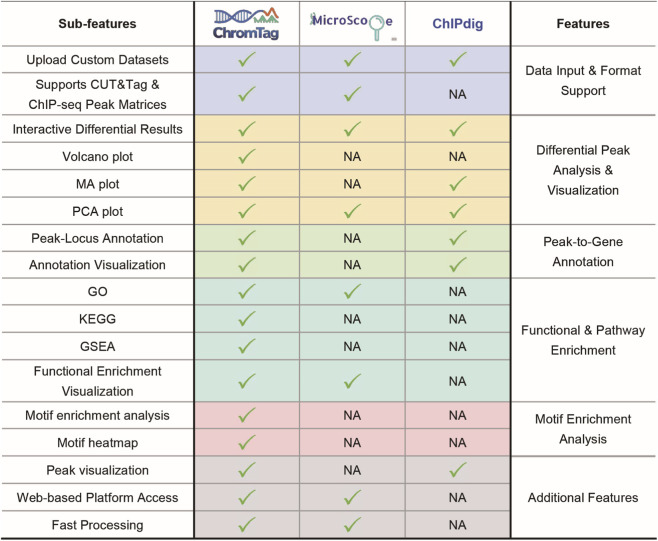
Comparison of ChromTag with other epigenomic analysis tools. “NA” indicates that the feature is not supported or not applicable for the tool, while a check mark denotes that the feature is available.

A detailed feature comparison among ChromTag, MicroScope, and ChIPdig is summarized in Figure 4. Collectively, these comparisons illustrate that ChromTag complements existing tools by providing a more integrated and interactive framework that connects preprocessed peak count matrices with downstream functional interpretation, facilitating comprehensive exploration of chromatin regulatory landscapes.

In addition to tools with similar functionality, ChromTag can also be positioned within a broader ecosystem of epigenomic analysis platforms. For example, deepTools primarily focuses on the processing and visualization of alignment-level data (e.g., BAM and bigWig files), while Galaxy provides a flexible workflow environment for modular and pipeline-based NGS data analysis (Ramírez et al., 2014; Afgan et al., 2018). In contrast, ChromTag emphasizes integrated downstream interpretation of peak-based results, offering a streamlined and interactive framework that connects differential analysis with functional annotation and biological interpretation. This complementary positioning highlights the role of ChromTag in bridging the gap between data processing and functional insight.

## 4 Conclusion

ChromTag is an interactive web-based application developed on the R Shiny framework to facilitate the comprehensive analysis of CUT&Tag and other epigenomic profiling datasets. By integrating differential peak detection, gene annotation, and multi-level enrichment analyses within a single platform, it streamlines downstream analysis from peak count matrices to functional interpretation, enabling users to uncover biologically meaningful chromatin alterations under diverse experimental conditions.

The visualization system provides a wide array of intuitive and customizable plots that support the examination of peak distributions, annotation outcomes, and pathway enrichment results. These features allow users to efficiently identify key pathways and functional modules associated with specific chromatin states, thereby deepening the understanding of epigenetic regulation across various biological contexts. Moreover, the Motif Enrichment Module extends the analytical scope of ChromTag by detecting overrepresented transcription factor binding motifs and revealing potential cooperative regulatory relationships. With its user-friendly interface and parameter flexibility, ChromTag accommodates both experimental biologists with limited computational experience and advanced users seeking greater analytical control.

Importantly, ChromTag is designed as an exploratory and integrative analysis platform. Appropriate statistical practices should be followed when interpreting results, including the use of sufficient biological replicates and the careful selection of filtering and significance thresholds based on data-driven considerations. Threshold parameters should not be adjusted *post hoc* to achieve statistical significance, as such practices may introduce bias. In addition, ChromTag adopts preprocessed peak count matrices as input, enabling efficient and responsive analysis within a web-based environment. This design avoids the computational burden associated with large alignment files (e.g., BAM files) and facilitates interactive exploration, making the platform suitable for real-time analysis and visualization.

Overall, ChromTag provides a practical, scalable, and biologically interpretable solution for integrative epigenomic research. The main contribution of ChromTag lies in the integration of downstream analysis steps into a unified and interactive workflow, addressing the gap between data processing and biological interpretation. We emphasize that ChromTag is designed to facilitate downstream analysis and interpretation, and that results should be evaluated in the context of underlying assumptions, data quality, and experimental design. Future developments will aim to extend its capabilities to single-cell and multi-omics datasets, further enhancing its utility for dissecting complex chromatin regulatory mechanisms.

## Data Availability

The ChIP-seq datasets used in this study are publicly available from the ENCODE project. H3K27ac datasets for A549 cells treated with dexamethasone for 0 h and 4 h were obtained from ENCODE experiments ENCSR783SNV (GEO: GSE91337) and ENCSR543ZVZ (GEO: GSE91282), respectively. All data are accessible through the ENCODE portal (https://www.encodeproject.org/) and the NCBI Gene Expression Omnibus (GEO, https://www.ncbi.nlm.nih.gov/geo/). The GitHub development page of ChromTag is https://github.com/fluquor1214/ChromTag
